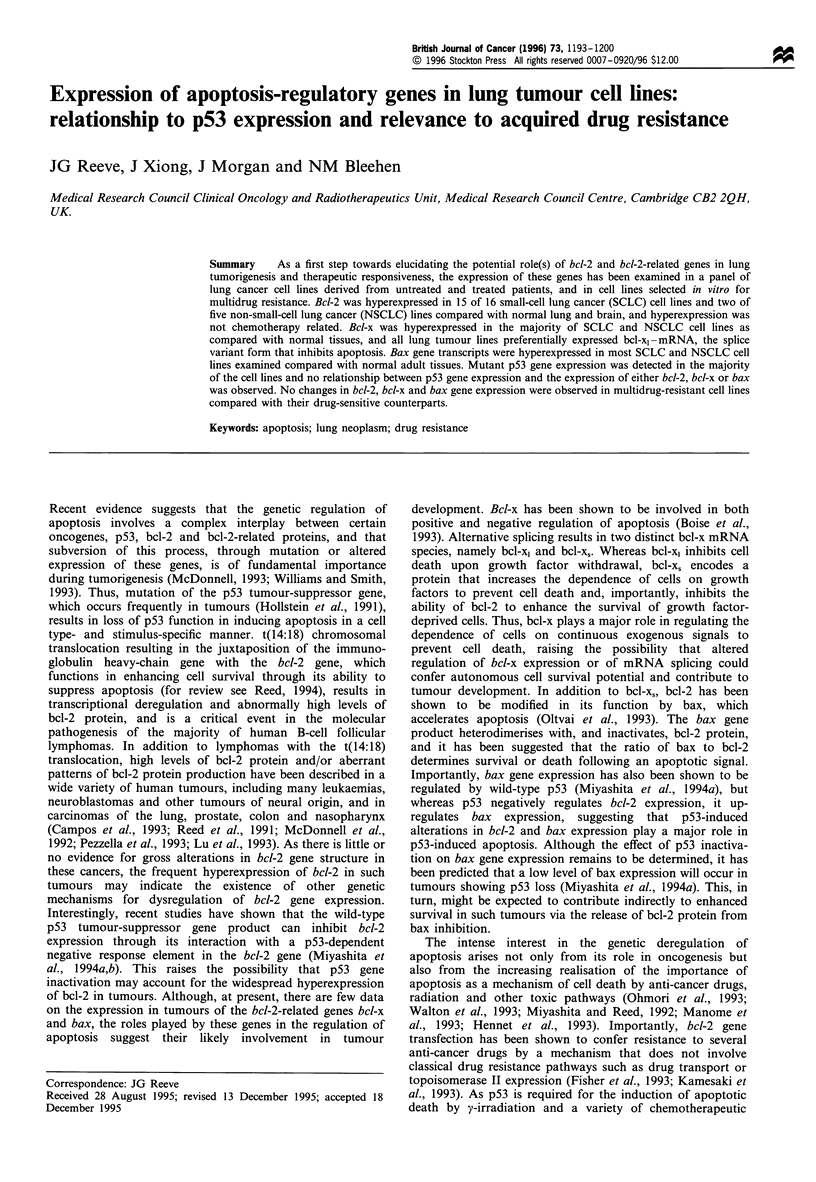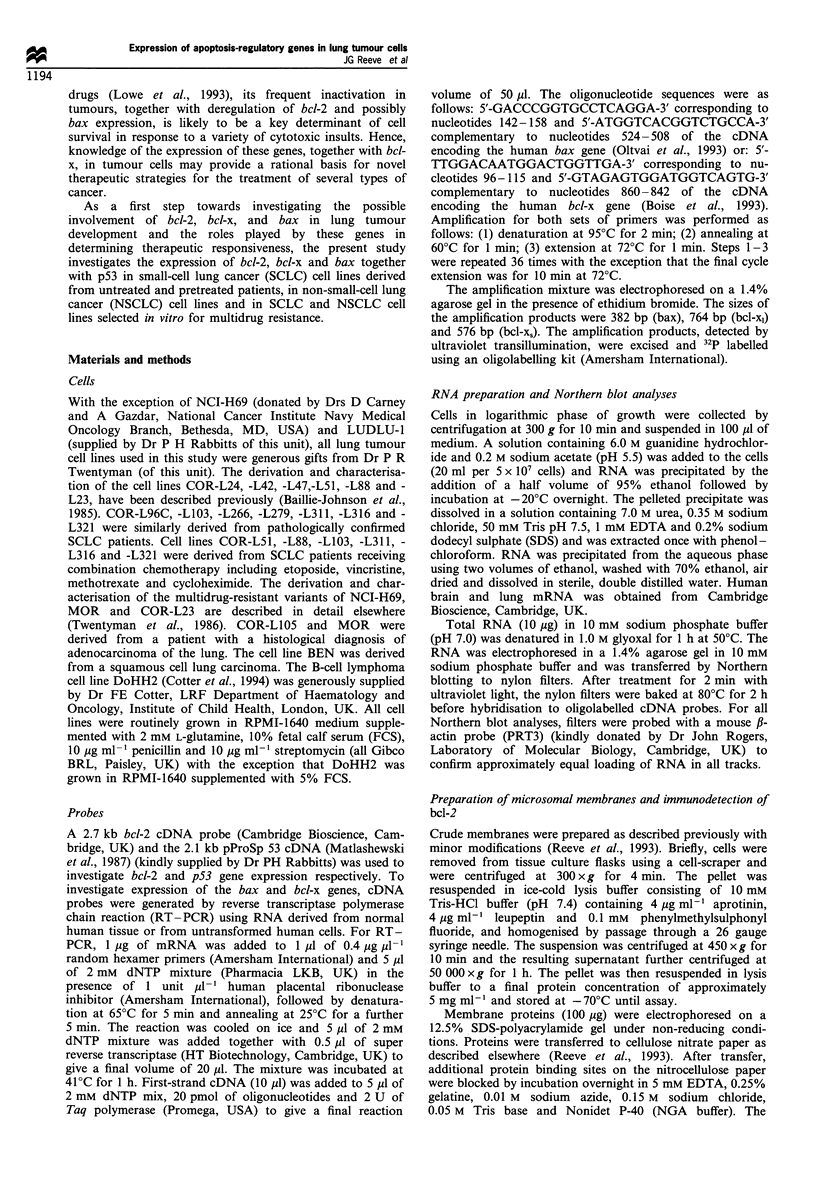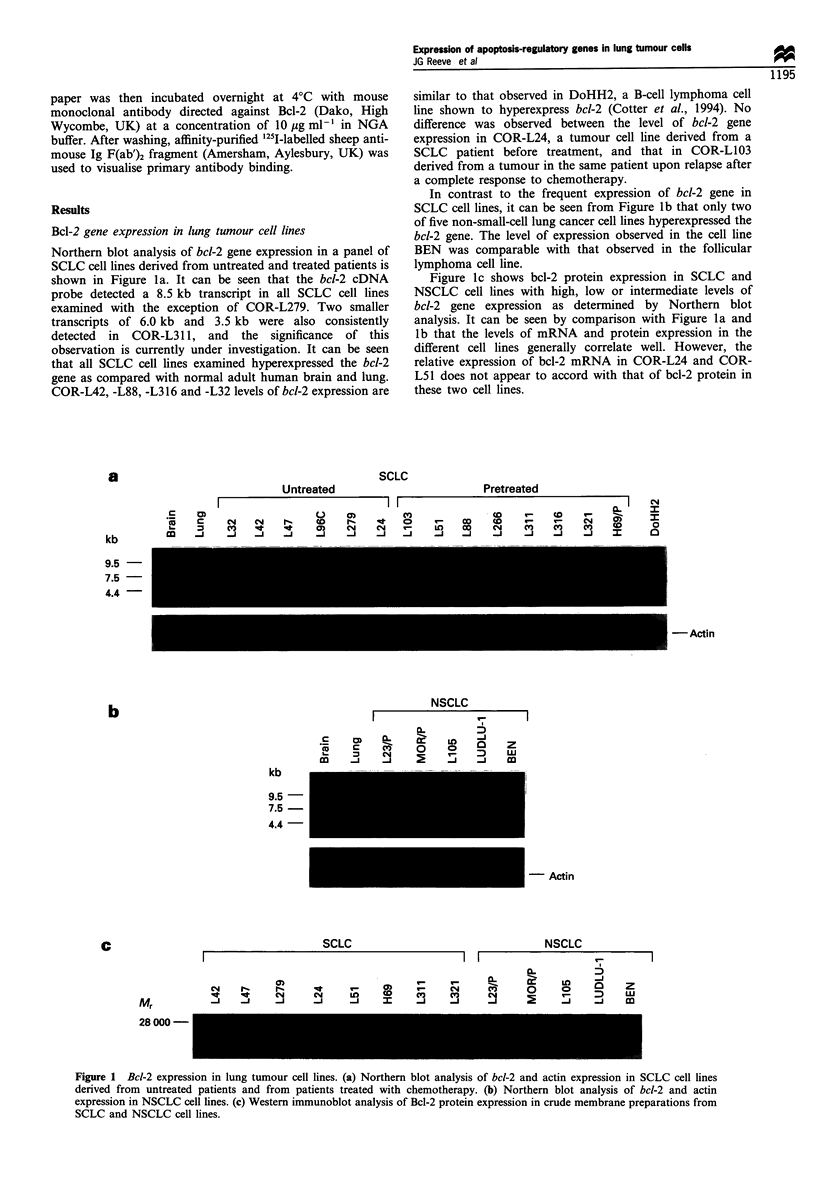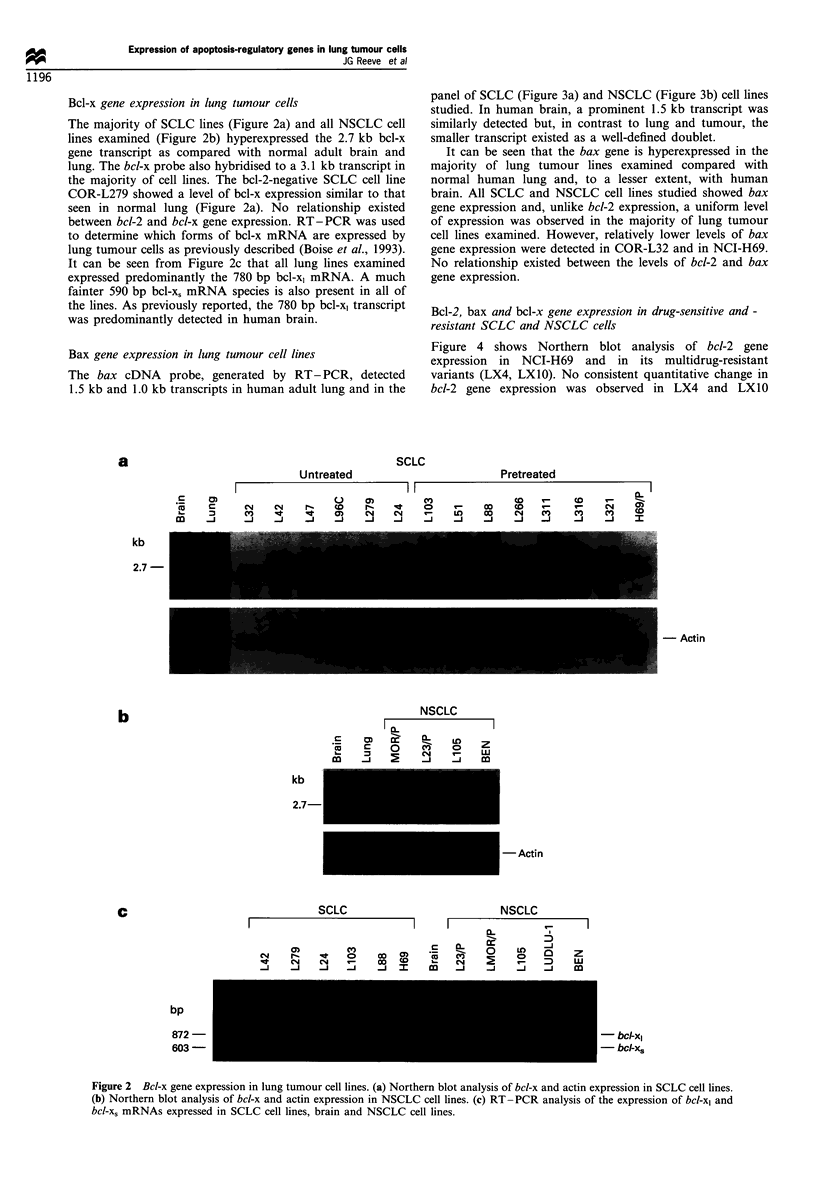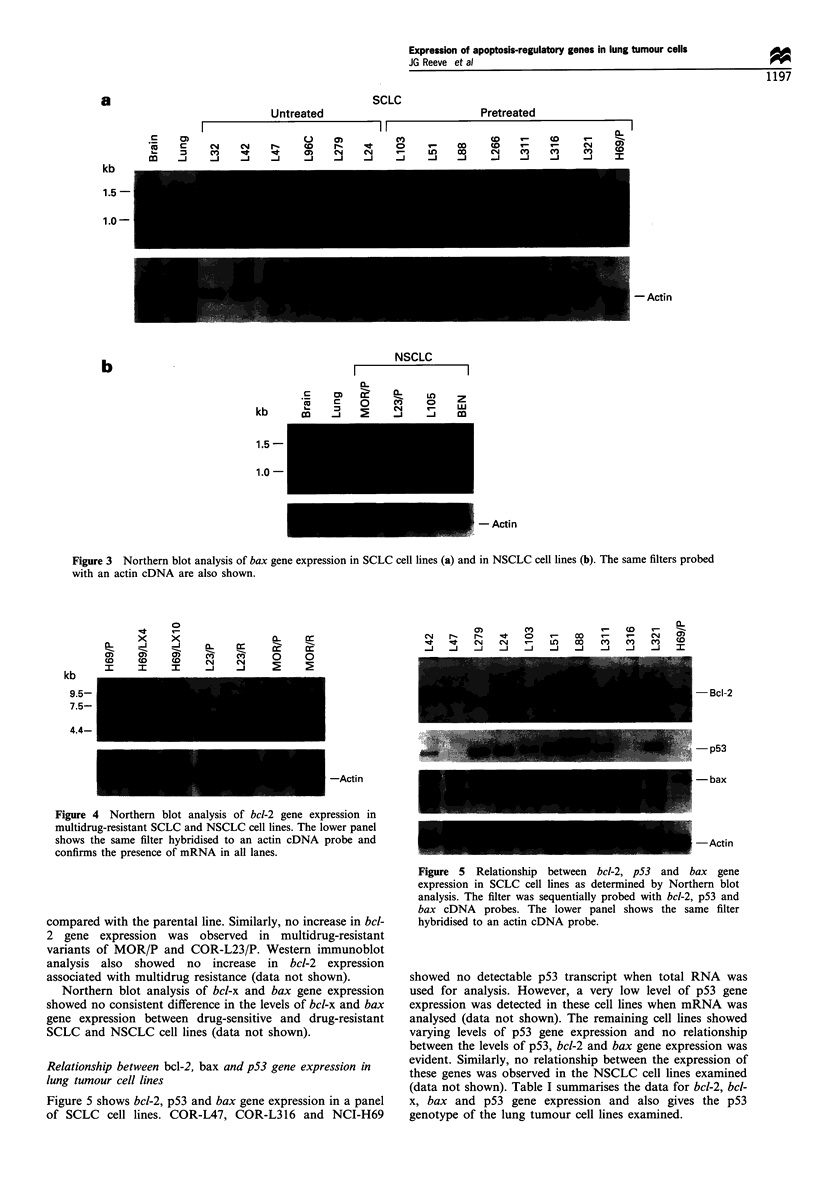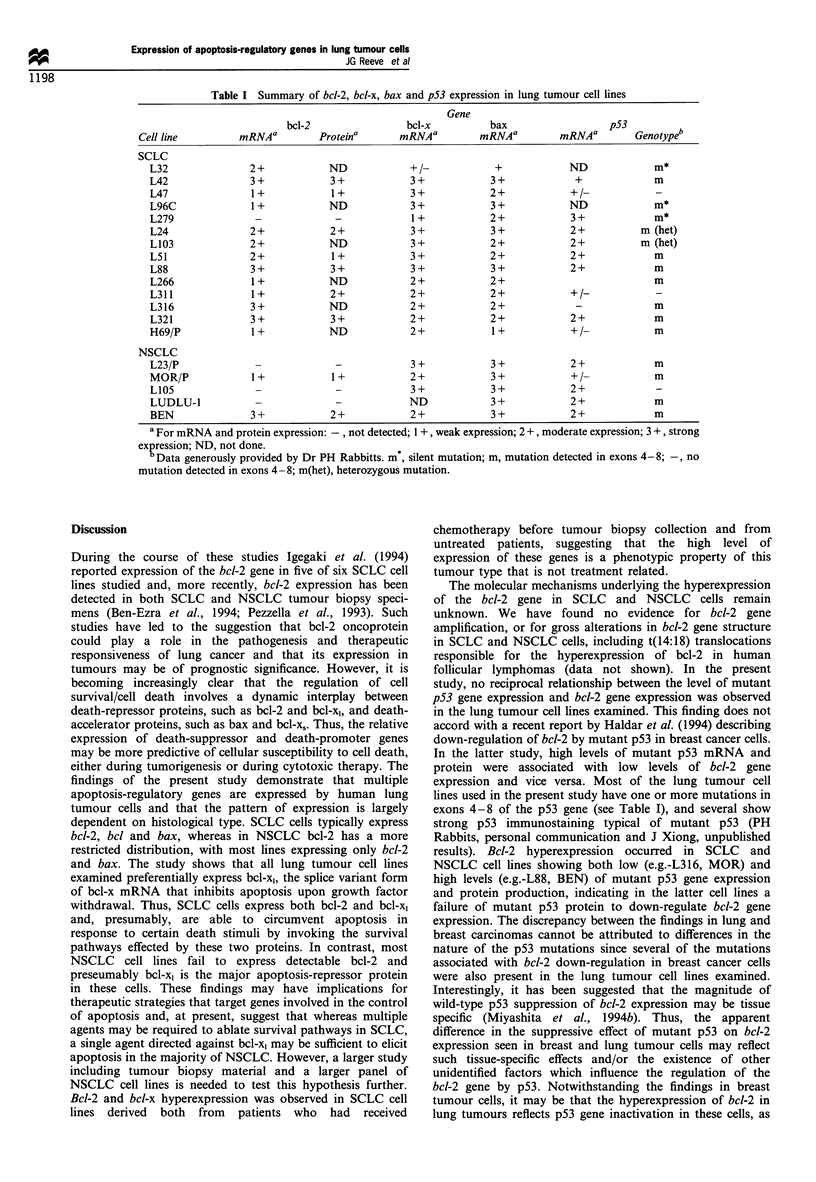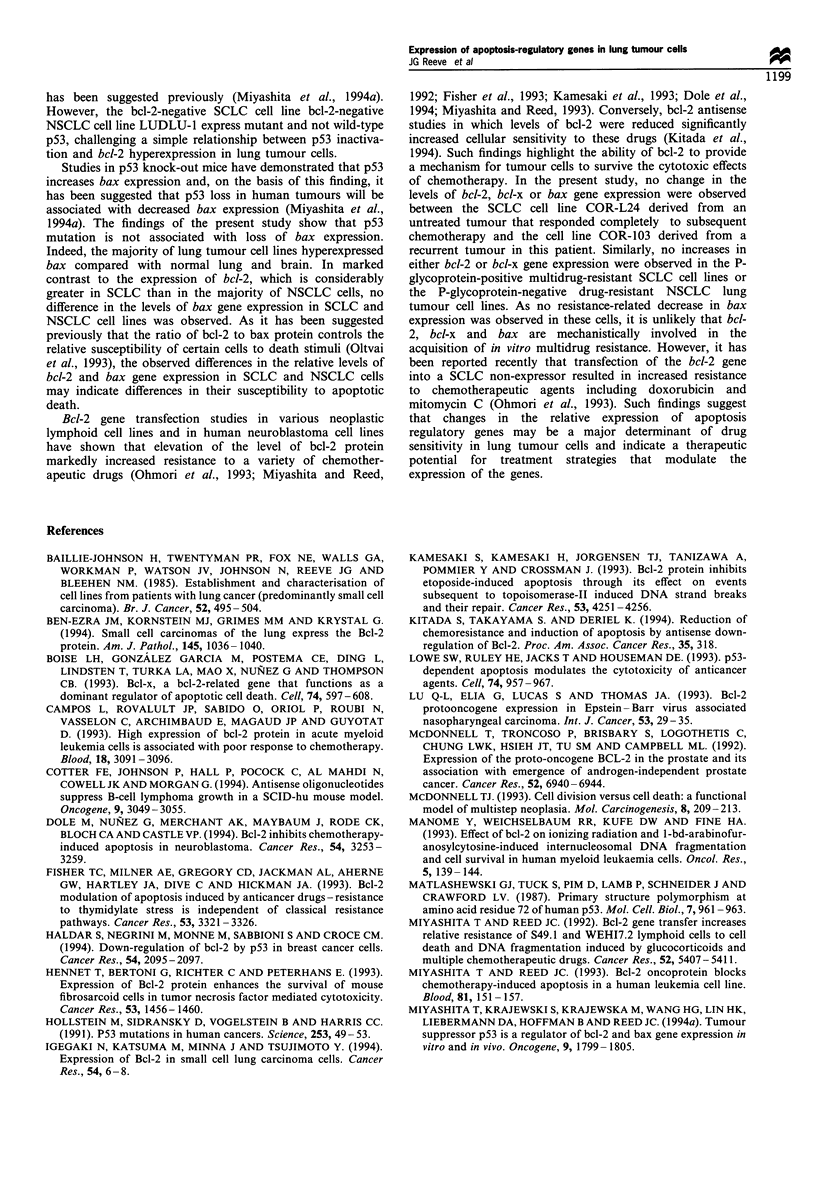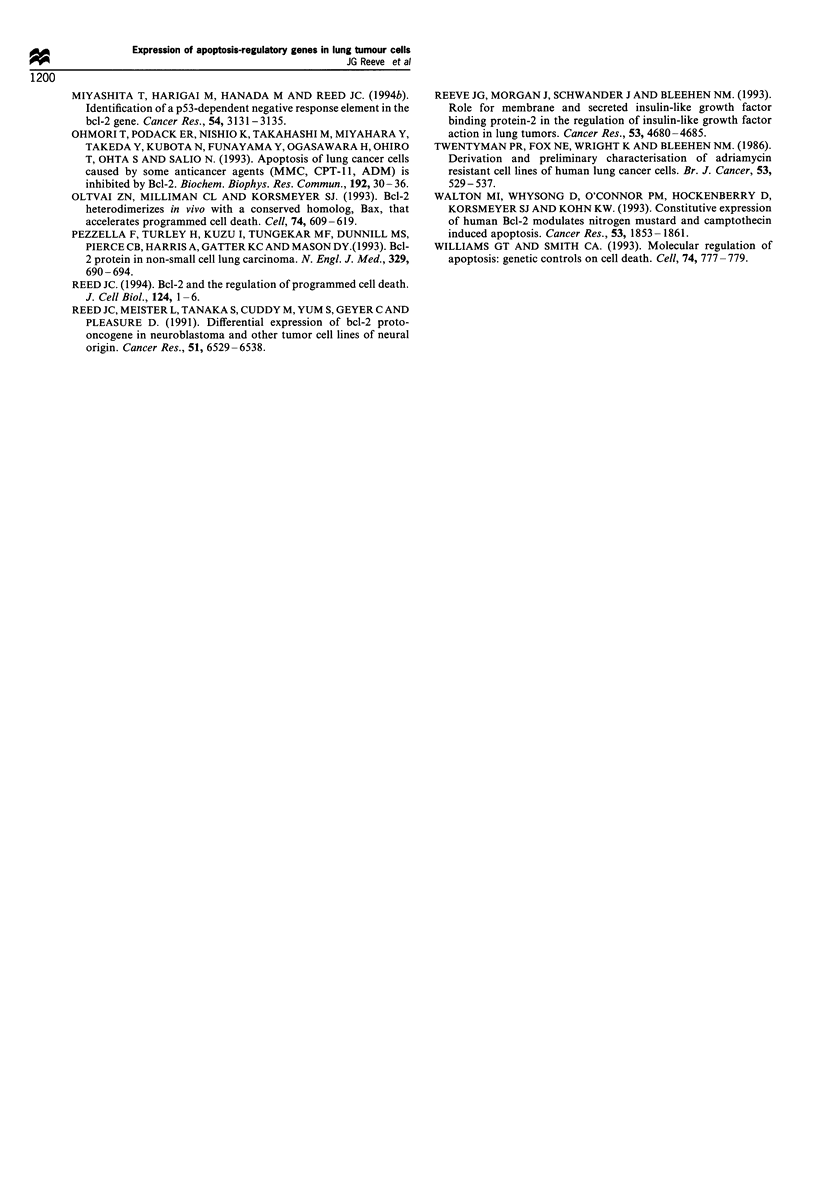# Expression of apoptosis-regulatory genes in lung tumour cell lines: relationship to p53 expression and relevance to acquired drug resistance.

**DOI:** 10.1038/bjc.1996.230

**Published:** 1996-05

**Authors:** J. G. Reeve, J. Xiong, J. Morgan, N. M. Bleehen

**Affiliations:** Medical Research Council Clinical Oncology and Radiotherapeutics Unit, Medical Research Council Centre, Cambridge, UK.

## Abstract

**Images:**


British Journal of Cancer (1996) 73, 1193-1200
© 1996 Stockton Press All rights reserved 0007-0920/96 $12.00

### Expression of apoptosis-regulatory genes in lung tumour cell lines: relationship to p53 expression and relevance to acquired drug resistance

JG Reeve, J Xiong, J Morgan and NM Bleehen

*Medical Research Council Clinical Oncology and Radiotherapeutics Unit, Medical Research Council Centre, Cambridge CB2 2QH, UK.*

**Summary** As a first step towards elucidating the potential role(s) of *bcl*-2 and *bcl*-2-related genes in lung tumorigenesis and therapeutic responsiveness, the expression of these genes has been examined in a panel of lung cancer cell lines derived from untreated and treated patients, and in cell lines selected *in vitro* for multidrug resistance. *Bcl*-2 was hyperexpressed in 15 of 16 small-cell lung cancer (SCLC) cell lines and two of five non-small-cell lung cancer (NSCLC) lines compared with normal lung and brain, and hyperexpression was not chemotherapy related. *Bcl*-x was hyperexpressed in the majority of SCLC and NSCLC cell lines as compared with normal tissues, and all lung tumour lines preferentially expressed bcl-$x_l$−mRNA, the splice variant form that inhibits apoptosis. *Bax* gene transcripts were hyperexpressed in most SCLC and NSCLC cell lines examined compared with normal adult tissues. Mutant p53 gene expression was detected in the majority of the cell lines and no relationship between p53 gene expression and the expression of either *bcl*-2, *bcl*-x or *bax* was observed. No changes in *bcl*-2, *bcl*-x and *bax* gene expression were observed in multidrug-resistant cell lines compared with their drug-sensitive counterparts.

**Keywords:** apoptosis; lung neoplasm; drug resistance

Recent evidence suggests that the genetic regulation of apoptosis involves a complex interplay between certain oncogenes, p53, bcl-2 and bcl-2-related proteins, and that subversion of this process, through mutation or altered expression of these genes, is of fundamental importance during tumorigenesis (McDonnell, 1993; Williams and Smith, 1993). Thus, mutation of the p53 tumour-suppressor gene, which occurs frequently in tumours (Hollstein *et al.*, 1991), results in loss of p53 function in inducing apoptosis in a cell type- and stimulus-specific manner. t(14:18) chromosomal translocation resulting in the juxtaposition of the immunoglobulin heavy-chain gene with the *bcl*-2 gene, which functions in enhancing cell survival through its ability to suppress apoptosis (for review see Reed, 1994), results in transcriptional deregulation and abnormally high levels of bcl-2 protein, and is a critical event in the molecular pathogenesis of the majority of human B-cell follicular lymphomas. In addition to lymphomas with the t(14:18) translocation, high levels of bcl-2 protein and/or aberrant patterns of bcl-2 protein production have been described in a wide variety of human tumours, including many leukaemias, neuroblastomas and other tumours of neural origin, and in carcinomas of the lung, prostate, colon and nasopharynx (Campos *et al.*, 1993; Reed *et al.*, 1991; McDonnell *et al.*, 1992; Pezzella *et al.*, 1993; Lu *et al.*, 1993). As there is little or no evidence for gross alterations in *bcl*-2 gene structure in these cancers, the frequent hyperexpression of *bcl*-2 in such tumours may indicate the existence of other genetic mechanisms for dysregulation of *bcl*-2 gene expression. Interestingly, recent studies have shown that the wild-type p53 tumour-suppressor gene product can inhibit *bcl*-2 expression through its interaction with a p53-dependent negative response element in the *bcl*-2 gene (Miyashita *et al.*, 1994*a*,*b*). This raises the possibility that p53 gene inactivation may account for the widespread hyperexpression of bcl-2 in tumours. Although, at present, there are few data on the expression in tumours of the *bcl*-2-related genes *bcl*-x and *bax*, the roles played by these genes in the regulation of apoptosis suggest their likely involvement in tumour development. *Bcl*-x has been shown to be involved in both positive and negative regulation of apoptosis (Boise *et al.*, 1993). Alternative splicing results in two distinct bcl-x mRNA species, namely bcl-$x_l$ and bcl-$x_s$. Whereas bcl-$x_l$ inhibits cell death upon growth factor withdrawal, bcl-$x_s$ encodes a protein that increases the dependence of cells on growth factors to prevent cell death and, importantly, inhibits the ability of bcl-2 to enhance the survival of growth factor-deprived cells. Thus, bcl-x plays a major role in regulating the dependence of cells on continuous exogenous signals to prevent cell death, raising the possibility that altered regulation of *bcl*-x expression or of mRNA splicing could confer autonomous cell survival potential and contribute to tumour development. In addition to bcl-$x_s$, bcl-2 has been shown to be modified in its function by bax, which accelerates apoptosis (Oltvai *et al.*, 1993). The *bax* gene product heterodimerises with, and inactivates, bcl-2 protein, and it has been suggested that the ratio of bax to bcl-2 determines survival or death following an apoptotic signal. Importantly, *bax* gene expression has also been shown to be regulated by wild-type p53 (Miyashita *et al.*, 1994*a*), but whereas p53 negatively regulates *bcl*-2 expression, it up-regulates *bax* expression, suggesting that p53-induced alterations in *bcl*-2 and *bax* expression play a major role in p53-induced apoptosis. Although the effect of p53 inactivation on *bax* gene expression remains to be determined, it has been predicted that a low level of bax expression will occur in tumours showing p53 loss (Miyashita *et al.*, 1994*a*). This, in turn, might be expected to contribute indirectly to enhanced survival in such tumours via the release of bcl-2 protein from bax inhibition.

The intense interest in the genetic deregulation of apoptosis arises not only from its role in oncogenesis but also from the increasing realisation of the importance of apoptosis as a mechanism of cell death by anti-cancer drugs, radiation and other toxic pathways (Ohmori *et al.*, 1993; Walton *et al.*, 1993; Miyashita and Reed, 1992; Manome *et al.*, 1993; Hennet *et al.*, 1993). Importantly, *bcl*-2 gene transfection has been shown to confer resistance to several anti-cancer drugs by a mechanism that does not involve classical drug resistance pathways such as drug transport or topoisomerase II expression (Fisher *et al.*, 1993; Kamesaki *et al.*, 1993). As p53 is required for the induction of apoptotic death by γ-irradiation and a variety of chemotherapeutic

Correspondence: JG Reeve
Received 28 August 1995; revised 13 December 1995; accepted 18 December 1995

drugs (Lowe *et al.*, 1993), its frequent inactivation in tumours, together with deregulation of *bcl*-2 and possibly *bax* expression, is likely to be a key determinant of cell survival in response to a variety of cytotoxic insults. Hence, knowledge of the expression of these genes, together with *bcl*-x, in tumour cells may provide a rational basis for novel therapeutic strategies for the treatment of several types of cancer.

As a first step towards investigating the possible involvement of *bcl*-2, *bcl*-x, and *bax* in lung tumour development and the roles played by these genes in determining therapeutic responsiveness, the present study investigates the expression of *bcl*-2, *bcl*-x and *bax* together with p53 in small-cell lung cancer (SCLC) cell lines derived from untreated and pretreated patients, in non-small-cell lung cancer (NSCLC) cell lines and in SCLC and NSCLC cell lines selected *in vitro* for multidrug resistance.

### Materials and methods

### *Cells*

With the exception of NCI-H69 (donated by Drs D Carney and A Gazdar, National Cancer Institute Navy Medical Oncology Branch, Bethesda, MD, USA) and LUDLU-1 (supplied by Dr P H Rabbitts of this unit), all lung tumour cell lines used in this study were generous gifts from Dr P R Twentyman (of this unit). The derivation and characterisation of the cell lines COR-L24, -L42, -L47,-L51, -L88 and -L23, have been described previously (Baillie-Johnson *et al.*, 1985). COR-L96C, -L103, -L266, -L279, -L311, -L316 and -L321 were similarly derived from pathologically confirmed SCLC patients. Cell lines COR-L51, -L88, -L103, -L311, -L316 and -L321 were derived from SCLC patients receiving combination chemotherapy including etoposide, vincristine, methotrexate and cycloheximide. The derivation and characterisation of the multidrug-resistant variants of NCI-H69, MOR and COR-L23 are described in detail elsewhere (Twentyman *et al.*, 1986). COR-L105 and MOR were derived from a patient with a histological diagnosis of adenocarcinoma of the lung. The cell line BEN was derived from a squamous cell lung carcinoma. The B-cell lymphoma cell line DoHH2 (Cotter *et al.*, 1994) was generously supplied by Dr FE Cotter, LRF Department of Haematology and Oncology, Institute of Child Health, London, UK. All cell lines were routinely grown in RPMI-1640 medium supplemented with 2 mM L-glutamine, 10% fetal calf serum (FCS), 10 $\mu g\ ml^{-1}$ penicillin and 10 $\mu g\ ml^{-1}$ streptomycin (all Gibco BRL, Paisley, UK) with the exception that DoHH2 was grown in RPMI-1640 supplemented with 5% FCS.

### *Probes*

A 2.7 kb *bcl*-2 cDNA probe (Cambridge Bioscience, Cambridge, UK) and the 2.1 kb pProSp 53 cDNA (Matlashewski *et al.*, 1987) (kindly supplied by Dr PH Rabbitts) was used to investigate *bcl*-2 and *p53* gene expression respectively. To investigate expression of the *bax* and *bcl*-x genes, cDNA probes were generated by reverse transcriptase polymerase chain reaction (RT–PCR) using RNA derived from normal human tissue or from untransformed human cells. For RT–PCR, 1 $\mu g$ of mRNA was added to 1 $\mu l$ of 0.4 $\mu g\ \mu l^{-1}$ random hexamer primers (Amersham International) and 5 $\mu l$ of 2 mM dNTP mixture (Pharmacia LKB, UK) in the presence of 1 unit $\mu l^{-1}$ human placental ribonuclease inhibitor (Amersham International), followed by denaturation at 65°C for 5 min and annealing at 25°C for a further 5 min. The reaction was cooled on ice and 5 $\mu l$ of 2 mM dNTP mixture was added together with 0.5 $\mu l$ of super reverse transcriptase (HT Biotechnology, Cambridge, UK) to give a final volume of 20 $\mu l$. The mixture was incubated at 41°C for 1 h. First-strand cDNA (10 $\mu l$) was added to 5 $\mu l$ of 2 mM dNTP mix, 20 pmol of oligonucleotides and 2 U of *Taq* polymerase (Promega, USA) to give a final reaction volume of 50 $\mu l$. The oligonucleotide sequences were as follows: 5′-GACCCGGTGCCTCAGGA-3′ corresponding to nucleotides 142–158 and 5′-ATGGTCACGGTCTGCCA-3′ complementary to nucleotides 524–508 of the cDNA encoding the human *bax* gene (Oltvai *et al.*, 1993) or: 5′-TTGGACAATGGACTGGTTGA-3′ corresponding to nucleotides 96–115 and 5′-GTAGAGTGGATGGTCAGTG-3′ complementary to nucleotides 860–842 of the cDNA encoding the human *bcl*-x gene (Boise *et al.*, 1993). Amplification for both sets of primers was performed as follows: (1) denaturation at 95°C for 2 min; (2) annealing at 60°C for 1 min; (3) extension at 72°C for 1 min. Steps 1–3 were repeated 36 times with the exception that the final cycle extension was for 10 min at 72°C.

The amplification mixture was electrophoresed on a 1.4% agarose gel in the presence of ethidium bromide. The sizes of the amplification products were 382 bp (bax), 764 bp ($bcl\text{-}x_l$) and 576 bp ($bcl\text{-}x_s$). The amplification products, detected by ultraviolet transillumination, were excised and $^{32}P$ labelled using an oligolabelling kit (Amersham International).

### *RNA preparation and Northern blot analyses*

Cells in logarithmic phase of growth were collected by centrifugation at 300 *g* for 10 min and suspended in 100 $\mu l$ of medium. A solution containing 6.0 M guanidine hydrochloride and 0.2 M sodium acetate (pH 5.5) was added to the cells (20 ml per $5 \times 10^7$ cells) and RNA was precipitated by the addition of a half volume of 95% ethanol followed by incubation at −20°C overnight. The pelleted precipitate was dissolved in a solution containing 7.0 M urea, 0.35 M sodium chloride, 50 mM Tris pH 7.5, 1 mM EDTA and 0.2% sodium dodecyl sulphate (SDS) and was extracted once with phenol–chloroform. RNA was precipitated from the aqueous phase using two volumes of ethanol, washed with 70% ethanol, air dried and dissolved in sterile, double distilled water. Human brain and lung mRNA was obtained from Cambridge Bioscience, Cambridge, UK.

Total RNA (10 $\mu g$) in 10 mM sodium phosphate buffer (pH 7.0) was denatured in 1.0 M glyoxal for 1 h at 50°C. The RNA was electrophoresed in a 1.4% agarose gel in 10 mM sodium phosphate buffer and was transferred by Northern blotting to nylon filters. After treatment for 2 min with ultraviolet light, the nylon filters were baked at 80°C for 2 h before hybridisation to oligolabelled cDNA probes. For all Northern blot analyses, filters were probed with a mouse $\beta$-actin probe (PRT3) (kindly donated by Dr John Rogers, Laboratory of Molecular Biology, Cambridge, UK) to confirm approximately equal loading of RNA in all tracks.

### *Preparation of microsomal membranes and immunodetection of* bcl-*2*

Crude membranes were prepared as described previously with minor modifications (Reeve *et al.*, 1993). Briefly, cells were removed from tissue culture flasks using a cell-scraper and were centrifuged at $300 \times g$ for 4 min. The pellet was resuspended in ice-cold lysis buffer consisting of 10 mM Tris-HCl buffer (pH 7.4) containing 4 $\mu g\ ml^{-1}$ aprotinin, 4 $\mu g\ ml^{-1}$ leupeptin and 0.1 mM phenylmethylsulphonyl fluoride, and homogenised by passage through a 26 gauge syringe needle. The suspension was centrifuged at $450 \times g$ for 10 min and the resulting supernatant further centrifuged at $50\,000 \times g$ for 1 h. The pellet was then resuspended in lysis buffer to a final protein concentration of approximately 5 mg $ml^{-1}$ and stored at −70°C until assay.

Membrane proteins (100 $\mu g$) were electrophoresed on a 12.5% SDS-polyacrylamide gel under non-reducing conditions. Proteins were transferred to cellulose nitrate paper as described elsewhere (Reeve *et al.*, 1993). After transfer, additional protein binding sites on the nitrocellulose paper were blocked by incubation overnight in 5 mM EDTA, 0.25% gelatine, 0.01 M sodium azide, 0.15 M sodium chloride, 0.05 M Tris base and Nonidet P-40 (NGA buffer). The paper was then incubated overnight at 4°C with mouse monoclonal antibody directed against Bcl-2 (Dako, High Wycombe, UK) at a concentration of 10 $\mu$g $ml^{-1}$ in NGA buffer. After washing, affinity-purified $^{125}$I-labelled sheep anti-mouse Ig $F(ab')_2$ fragment (Amersham, Aylesbury, UK) was used to visualise primary antibody binding.

### Results

*Bcl-2 gene expression in lung tumour cell lines*

Northern blot analysis of *bcl*-2 gene expression in a panel of SCLC cell lines derived from untreated and treated patients is shown in Figure 1a. It can be seen that the *bcl*-2 cDNA probe detected a 8.5 kb transcript in all SCLC cell lines examined with the exception of COR-L279. Two smaller transcripts of 6.0 kb and 3.5 kb were also consistently detected in COR-L311, and the significance of this observation is currently under investigation. It can be seen that all SCLC cell lines examined hyperexpressed the *bcl*-2 gene as compared with normal adult human brain and lung. COR-L42, -L88, -L316 and -L32 levels of *bcl*-2 expression are similar to that observed in DoHH2, a B-cell lymphoma cell line shown to hyperexpress *bcl*-2 (Cotter *et al.*, 1994). No difference was observed between the level of *bcl*-2 gene expression in COR-L24, a tumour cell line derived from a SCLC patient before treatment, and that in COR-L103 derived from a tumour in the same patient upon relapse after a complete response to chemotherapy.

In contrast to the frequent expression of *bcl*-2 gene in SCLC cell lines, it can be seen from Figure 1b that only two of five non-small-cell lung cancer cell lines hyperexpressed the *bcl*-2 gene. The level of expression observed in the cell line BEN was comparable with that observed in the follicular lymphoma cell line.

Figure 1c shows bcl-2 protein expression in SCLC and NSCLC cell lines with high, low or intermediate levels of *bcl*-2 gene expression as determined by Northern blot analysis. It can be seen by comparison with Figure 1a and 1b that the levels of mRNA and protein expression in the different cell lines generally correlate well. However, the relative expression of bcl-2 mRNA in COR-L24 and COR-L51 does not appear to accord with that of bcl-2 protein in these two cell lines.

**Figure 1** *Bcl*-2 expression in lung tumour cell lines. (**a**) Northern blot analysis of *bcl*-2 and actin expression in SCLC cell lines derived from untreated patients and from patients treated with chemotherapy. (**b**) Northern blot analysis of *bcl*-2 and actin expression in NSCLC cell lines. (**c**) Western immunoblot analysis of Bcl-2 protein expression in crude membrane preparations from SCLC and NSCLC cell lines.

### Bcl-x *gene expression in lung tumour cells*

The majority of SCLC lines (Figure 2a) and all NSCLC cell lines examined (Figure 2b) hyperexpressed the 2.7 kb bcl-x gene transcript as compared with normal adult brain and lung. The *bcl*-x probe also hybridised to a 3.1 kb transcript in the majority of cell lines. The bcl-2-negative SCLC cell line COR-L279 showed a level of bcl-x expression similar to that seen in normal lung (Figure 2a). No relationship existed between *bcl*-2 and *bcl*-x gene expression. RT–PCR was used to determine which forms of bcl-x mRNA are expressed by lung tumour cells as previously described (Boise *et al.*, 1993). It can be seen from Figure 2c that all lung lines examined expressed predominantly the 780 bp bcl-$x_l$ mRNA. A much fainter 590 bp bcl-$x_s$ mRNA species is also present in all of the lines. As previously reported, the 780 bp bcl-$x_l$ transcript was predominantly detected in human brain.

### Bax *gene expression in lung tumour cell lines*

The *bax* cDNA probe, generated by RT–PCR, detected 1.5 kb and 1.0 kb transcripts in human adult lung and in the panel of SCLC (Figure 3a) and NSCLC (Figure 3b) cell lines studied. In human brain, a prominent 1.5 kb transcript was similarly detected but, in contrast to lung and tumour, the smaller transcript existed as a well-defined doublet.

It can be seen that the *bax* gene is hyperexpressed in the majority of lung tumour lines examined compared with normal human lung and, to a lesser extent, with human brain. All SCLC and NSCLC cell lines studied showed *bax* gene expression and, unlike *bcl*-2 expression, a uniform level of expression was observed in the majority of lung tumour cell lines examined. However, relatively lower levels of *bax* gene expression were detected in COR-L32 and in NCI-H69. No relationship existed between the levels of *bcl*-2 and *bax* gene expression.

### Bcl-*2,* bax *and* bcl-*x gene expression in drug-sensitive and -resistant SCLC and NSCLC cells*

Figure 4 shows Northern blot analysis of *bcl*-2 gene expression in NCI-H69 and in its multidrug-resistant variants (LX4, LX10). No consistent quantitative change in *bcl*-2 gene expression was observed in LX4 and LX10

**Figure 2** *Bcl*-x gene expression in lung tumour cell lines. (**a**) Northern blot analysis of *bcl*-x and actin expression in SCLC cell lines. (**b**) Northern blot analysis of *bcl*-x and actin expression in NSCLC cell lines. (**c**) RT–PCR analysis of the expression of *bcl*-$x_l$ and *bcl*-$x_s$ mRNAs expressed in SCLC cell lines, brain and NSCLC cell lines.

**Figure 3** Northern blot analysis of *bax* gene expression in SCLC cell lines (**a**) and in NSCLC cell lines (**b**). The same filters probed with an actin cDNA are also shown.

**Figure 4** Northern blot analysis of *bcl*-2 gene expression in multidrug-resistant SCLC and NSCLC cell lines. The lower panel shows the same filter hybridised to an actin cDNA probe and confirms the presence of mRNA in all lanes.

**Figure 5** Relationship between *bcl*-2, *p53* and *bax* gene expression in SCLC cell lines as determined by Northern blot analysis. The filter was sequentially probed with *bcl*-2, p53 and *bax* cDNA probes. The lower panel shows the same filter hybridised to an actin cDNA probe.

compared with the parental line. Similarly, no increase in *bcl*-2 gene expression was observed in multidrug-resistant variants of MOR/P and COR-L23/P. Western immunoblot analysis also showed no increase in *bcl*-2 expression associated with multidrug resistance (data not shown).

Northern blot analysis of *bcl*-x and *bax* gene expression showed no consistent difference in the levels of *bcl*-x and *bax* gene expression between drug-sensitive and drug-resistant SCLC and NSCLC cell lines (data not shown).

### *Relationship between* bcl-2, bax *and p53 gene expression in lung tumour cell lines*

Figure 5 shows *bcl*-2, p53 and *bax* gene expression in a panel of SCLC cell lines. COR-L47, COR-L316 and NCI-H69 showed no detectable p53 transcript when total RNA was used for analysis. However, a very low level of p53 gene expression was detected in these cell lines when mRNA was analysed (data not shown). The remaining cell lines showed varying levels of p53 gene expression and no relationship between the levels of p53, *bcl*-2 and *bax* gene expression was evident. Similarly, no relationship between the expression of these genes was observed in the NSCLC cell lines examined (data not shown). Table I summarises the data for *bcl*-2, *bcl*-x, *bax* and p53 gene expression and also gives the p53 genotype of the lung tumour cell lines examined.

**Table I** Summary of *bcl*-2, *bcl*-x, *bax* and *p53* expression in lung tumour cell lines

| | *Gene* | | | | | |
|---|---|---|---|---|---|---|
| | bcl-*2* | | bcl-*x* | bax | *p53* | |
| *Cell line* | *mRNA*[a] | *Protein*[a] | *mRNA*[a] | *mRNA*[a] | *mRNA*[a] | *Genotype*[b] |
| SCLC | | | | | | |
| L32 | 2+ | ND | +/– | + | ND | m* |
| L42 | 3+ | 3+ | 3+ | 3+ | + | m |
| L47 | 1+ | 1+ | 3+ | 2+ | +/– | – |
| L96C | 1+ | ND | 3+ | 3+ | ND | m* |
| L279 | – | – | 1+ | 2+ | 3+ | m* |
| L24 | 2+ | 2+ | 3+ | 3+ | 2+ | m (het) |
| L103 | 2+ | ND | 3+ | 2+ | 2+ | m (het) |
| L51 | 2+ | 1+ | 3+ | 2+ | 2+ | m |
| L88 | 3+ | 3+ | 3+ | 3+ | 2+ | m |
| L266 | 1+ | ND | 2+ | 2+ | | m |
| L311 | 1+ | 2+ | 2+ | 2+ | +/– | – |
| L316 | 3+ | ND | 2+ | 2+ | – | m |
| L321 | 3+ | 3+ | 2+ | 2+ | 2+ | m |
| H69/P | 1+ | ND | 2+ | 1+ | +/– | m |
| NSCLC | | | | | | |
| L23/P | – | – | 3+ | 3+ | 2+ | m |
| MOR/P | 1+ | 1+ | 2+ | 3+ | +/– | m |
| L105 | – | – | 3+ | 3+ | 2+ | – |
| LUDLU-1 | – | – | ND | 3+ | 2+ | m |
| BEN | 3+ | 2+ | 2+ | 3+ | 2+ | m |

[a] For mRNA and protein expression: –, not detected; 1+, weak expression; 2+, moderate expression; 3+, strong expression; ND, not done.
[b] Data generously provided by Dr PH Rabbitts. m*, silent mutation; m, mutation detected in exons 4–8; –, no mutation detected in exons 4–8; m(het), heterozygous mutation.

### Discussion

During the course of these studies Igegaki *et al.* (1994) reported expression of the *bcl*-2 gene in five of six SCLC cell lines studied and, more recently, *bcl*-2 expression has been detected in both SCLC and NSCLC tumour biopsy specimens (Ben-Ezra *et al.*, 1994; Pezzella *et al.*, 1993). Such studies have led to the suggestion that bcl-2 oncoprotein could play a role in the pathogenesis and therapeutic responsiveness of lung cancer and that its expression in tumours may be of prognostic significance. However, it is becoming increasingly clear that the regulation of cell survival/cell death involves a dynamic interplay between death-repressor proteins, such as bcl-2 and bcl-$x_l$, and death-accelerator proteins, such as bax and bcl-$x_s$. Thus, the relative expression of death-suppressor and death-promoter genes may be more predictive of cellular susceptibility to cell death, either during tumorigenesis or during cytotoxic therapy. The findings of the present study demonstrate that multiple apoptosis-regulatory genes are expressed by human lung tumour cells and that the pattern of expression is largely dependent on histological type. SCLC cells typically express *bcl*-2, *bcl* and *bax*, whereas in NSCLC bcl-2 has a more restricted distribution, with most lines expressing only *bcl*-2 and *bax*. The study shows that all lung tumour cell lines examined preferentially express bcl-$x_l$, the splice variant form of bcl-x mRNA that inhibits apoptosis upon growth factor withdrawal. Thus, SCLC cells express both bcl-2 and bcl-$x_l$ and, presumably, are able to circumvent apoptosis in response to certain death stimuli by invoking the survival pathways effected by these two proteins. In contrast, most NSCLC cell lines fail to express detectable bcl-2 and preseumably bcl-$x_l$ is the major apoptosis-repressor protein in these cells. These findings may have implications for therapeutic strategies that target genes involved in the control of apoptosis and, at present, suggest that whereas multiple agents may be required to ablate survival pathways in SCLC, a single agent directed against bcl-$x_l$ may be sufficient to elicit apoptosis in the majority of NSCLC. However, a larger study including tumour biopsy material and a larger panel of NSCLC cell lines is needed to test this hypothesis further. *Bcl*-2 and *bcl*-x hyperexpression was observed in SCLC cell lines derived both from patients who had received chemotherapy before tumour biopsy collection and from untreated patients, suggesting that the high level of expression of these genes is a phenotypic property of this tumour type that is not treatment related.

The molecular mechanisms underlying the hyperexpression of the *bcl*-2 gene in SCLC and NSCLC cells remain unknown. We have found no evidence for *bcl*-2 gene amplification, or for gross alterations in *bcl*-2 gene structure in SCLC and NSCLC cells, including t(14:18) translocations responsible for the hyperexpression of bcl-2 in human follicular lymphomas (data not shown). In the present study, no reciprocal relationship between the level of mutant *p53* gene expression and *bcl*-2 gene expression was observed in the lung tumour cell lines examined. This finding does not accord with a recent report by Haldar *et al.* (1994) describing down-regulation of *bcl*-2 by mutant p53 in breast cancer cells. In the latter study, high levels of mutant p53 mRNA and protein were associated with low levels of *bcl*-2 gene expression and vice versa. Most of the lung tumour cell lines used in the present study have one or more mutations in exons 4–8 of the p53 gene (see Table I), and several show strong p53 immunostaining typical of mutant p53 (PH Rabbits, personal communication and J Xiong, unpublished results). *Bcl*-2 hyperexpression occurred in SCLC and NSCLC cell lines showing both low (e.g.-L316, MOR) and high levels (e.g.-L88, BEN) of mutant p53 gene expression and protein production, indicating in the latter cell lines a failure of mutant p53 protein to down-regulate *bcl*-2 gene expression. The discrepancy between the findings in lung and breast carcinomas cannot be attributed to differences in the nature of the p53 mutations since several of the mutations associated with *bcl*-2 down-regulation in breast cancer cells were also present in the lung tumour cell lines examined. Interestingly, it has been suggested that the magnitude of wild-type p53 suppression of *bcl*-2 expression may be tissue specific (Miyashita *et al.*, 1994*b*). Thus, the apparent difference in the suppressive effect of mutant p53 on *bcl*-2 expression seen in breast and lung tumour cells may reflect such tissue-specific effects and/or the existence of other unidentified factors which influence the regulation of the *bcl*-2 gene by p53. Notwithstanding the findings in breast tumour cells, it may be that the hyperexpression of *bcl*-2 in lung tumours reflects p53 gene inactivation in these cells, as has been suggested previously (Miyashita *et al.*, 1994*a*). However, the bcl-2-negative SCLC cell line bcl-2-negative NSCLC cell line LUDLU-1 express mutant and not wild-type p53, challenging a simple relationship between p53 inactivation and *bcl*-2 hyperexpression in lung tumour cells.

Studies in p53 knock-out mice have demonstrated that p53 increases *bax* expression and, on the basis of this finding, it has been suggested that p53 loss in human tumours will be associated with decreased *bax* expression (Miyashita *et al.*, 1994*a*). The findings of the present study show that p53 mutation is not associated with loss of *bax* expression. Indeed, the majority of lung tumour cell lines hyperexpressed *bax* compared with normal lung and brain. In marked contrast to the expression of *bcl*-2, which is considerably greater in SCLC than in the majority of NSCLC cells, no difference in the levels of *bax* gene expression in SCLC and NSCLC cell lines was observed. As it has been suggested previously that the ratio of bcl-2 to bax protein controls the relative susceptibility of certain cells to death stimuli (Oltvai *et al.*, 1993), the observed differences in the relative levels of *bcl*-2 and *bax* gene expression in SCLC and NSCLC cells may indicate differences in their susceptibility to apoptotic death.

*Bcl*-2 gene transfection studies in various neoplastic lymphoid cell lines and in human neuroblastoma cell lines have shown that elevation of the level of bcl-2 protein markedly increased resistance to a variety of chemotherapeutic drugs (Ohmori *et al.*, 1993; Miyashita and Reed, 1992; Fisher *et al.*, 1993; Kamesaki *et al.*, 1993; Dole *et al.*, 1994; Miyashita and Reed, 1993). Conversely, bcl-2 antisense studies in which levels of bcl-2 were reduced significantly increased cellular sensitivity to these drugs (Kitada *et al.*, 1994). Such findings highlight the ability of bcl-2 to provide a mechanism for tumour cells to survive the cytotoxic effects of chemotherapy. In the present study, no change in the levels of *bcl*-2, *bcl*-x or *bax* gene expression were observed between the SCLC cell line COR-L24 derived from an untreated tumour that responded completely to subsequent chemotherapy and the cell line COR-103 derived from a recurrent tumour in this patient. Similarly, no increases in either *bcl*-2 or *bcl*-x gene expression were observed in the P-glycoprotein-positive multidrug-resistant SCLC cell lines or the P-glycoprotein-negative drug-resistant NSCLC lung tumour cell lines. As no resistance-related decrease in *bax* expression was observed in these cells, it is unlikely that *bcl*-2, *bcl*-x and *bax* are mechanistically involved in the acquisition of *in vitro* multidrug resistance. However, it has been reported recently that transfection of the *bcl*-2 gene into a SCLC non-expressor resulted in increased resistance to chemotherapeutic agents including doxorubicin and mitomycin C (Ohmori *et al.*, 1993). Such findings suggest that changes in the relative expression of apoptosis regulatory genes may be a major determinant of drug sensitivity in lung tumour cells and indicate a therapeutic potential for treatment strategies that modulate the expression of the genes.

### References

BAILLIE-JOHNSON H, TWENTYMAN PR, FOX NE, WALLS GA, WORKMAN P, WATSON JV, JOHNSON N, REEVE JG AND BLEEHEN NM. (1985). Establishment and characterisation of cell lines from patients with lung cancer (predominantly small cell carcinoma). *Br. J. Cancer*, **52**, 495–504.

BEN-EZRA JM, KORNSTEIN MJ, GRIMES MM AND KRYSTAL G. (1994). Small cell carcinomas of the lung express the Bcl-2 protein. *Am. J. Pathol.*, **145**, 1036–1040.

BOISE LH, GONZÁLEZ GARCIA M, POSTEMA CE, DING L, LINDSTEN T, TURKA LA, MAO X, NUÑEZ G AND THOMPSON CB. (1993). Bcl-x, a bcl-2-related gene that functions as a dominant regulator of apoptotic cell death. *Cell*, **74**, 597–608.

CAMPOS L, ROVALULT JP, SABIDO O, ORIOL P, ROUBI N, VASSELON C, ARCHIMBAUD E, MAGAUD JP AND GUYOTAT D. (1993). High expression of bcl-2 protein in acute myeloid leukemia cells is associated with poor response to chemotherapy. *Blood*, **18**, 3091–3096.

COTTER FE, JOHNSON P, HALL P, POCOCK C, AL MAHDI N, COWELL JK AND MORGAN G. (1994). Antisense oligonucleotides suppress B-cell lymphoma growth in a SCID-hu mouse model. *Oncogene*, **9**, 3049–3055.

DOLE M, NUÑEZ G, MERCHANT AK, MAYBAUM J, RODE CK, BLOCH CA AND CASTLE VP. (1994). Bcl-2 inhibits chemotherapy-induced apoptosis in neuroblastoma. *Cancer Res.*, **54**, 3253–3259.

FISHER TC, MILNER AE, GREGORY CD, JACKMAN AL, AHERNE GW, HARTLEY JA, DIVE C AND HICKMAN JA. (1993). Bcl-2 modulation of apoptosis induced by anticancer drugs–resistance to thymidylate stress is independent of classical resistance pathways. *Cancer Res.*, **53**, 3321–3326.

HALDAR S, NEGRINI M, MONNE M, SABBIONI S AND CROCE CM. (1994). Down-regulation of bcl-2 by p53 in breast cancer cells. *Cancer Res.*, **54**, 2095–2097.

HENNET T, BERTONI G, RICHTER C AND PETERHANS E. (1993). Expression of Bcl-2 protein enhances the survival of mouse fibrosarcoid cells in tumor necrosis factor mediated cytotoxicity. *Cancer Res.*, **53**, 1456–1460.

HOLLSTEIN M, SIDRANSKY D, VOGELSTEIN B AND HARRIS CC. (1991). P53 mutations in human cancers. *Science*, **253**, 49–53.

IGEGAKI N, KATSUMA M, MINNA J AND TSUJIMOTO Y. (1994). Expression of Bcl-2 in small cell lung carcinoma cells. *Cancer Res.*, **54**, 6–8.

KAMESAKI S, KAMESAKI H, JORGENSEN TJ, TANIZAWA A, POMMIER Y AND CROSSMAN J. (1993). Bcl-2 protein inhibits etoposide-induced apoptosis through its effect on events subsequent to topoisomerase-II induced DNA strand breaks and their repair. *Cancer Res.*, **53**, 4251–4256.

KITADA S, TAKAYAMA S. AND DERIEL K. (1994). Reduction of chemoresistance and induction of apoptosis by antisense down-regulation of Bcl-2. *Proc. Am. Assoc. Cancer Res.*, **35**, 318.

LOWE SW, RULEY HE, JACKS T AND HOUSEMAN DE. (1993). p53-dependent apoptosis modulates the cytotoxicity of anticancer agents. *Cell*, **74**, 957–967.

LU Q-L, ELIA G, LUCAS S AND THOMAS JA. (1993). Bcl-2 protooncogene expression in Epstein–Barr virus associated nasopharyngeal carcinoma. *Int. J. Cancer*, **53**, 29–35.

MCDONNELL T, TRONCOSO P, BRISBARY S, LOGOTHETIS C, CHUNG LWK, HSIEH JT, TU SM AND CAMPBELL ML. (1992). Expression of the proto-oncogene BCL-2 in the prostate and its association with emergence of androgen-independent prostate cancer. *Cancer Res.*, **52**, 6940–6944.

MCDONNELL TJ. (1993). Cell division versus cell death: a functional model of multistep neoplasia. *Mol. Carcinogenesis*, **8**, 209–213.

MANOME Y, WEICHSELBAUM RR, KUFE DW AND FINE HA. (1993). Effect of bcl-2 on ionizing radiation and 1-bd-arabinofuranosylcytosine-induced internucleosomal DNA fragmentation and cell survival in human myeloid leukaemia cells. *Oncol. Res.*, **5**, 139–144.

MATLASHEWSKI GJ, TUCK S, PIM D, LAMB P, SCHNEIDER J AND CRAWFORD LV. (1987). Primary structure polymorphism at amino acid residue 72 of human p53. *Mol. Cell. Biol.*, **7**, 961–963.

MIYASHITA T AND REED JC. (1992). Bcl-2 gene transfer increases relative resistance of S49.1 and WEHI7.2 lymphoid cells to cell death and DNA fragmentation induced by glucocorticoids and multiple chemotherapeutic drugs. *Cancer Res.*, **52**, 5407–5411.

MIYASHITA T AND REED JC. (1993). Bcl-2 oncoprotein blocks chemotherapy-induced apoptosis in a human leukemia cell line. *Blood*, **81**, 151–157.

MIYASHITA T, KRAJEWSKI S, KRAJEWSKA M, WANG HG, LIN HK, LIEBERMANN DA, HOFFMAN B AND REED JC. (1994*a*). Tumour suppressor p53 is a regulator of bcl-2 and bax gene expression *in vitro* and *in vivo*. *Oncogene*, **9**, 1799–1805.

MIYASHITA T, HARIGAI M, HANADA M AND REED JC. (1994*b*). Identification of a p53-dependent negative response element in the bcl-2 gene. *Cancer Res.*, **54**, 3131–3135.

OHMORI T, PODACK ER, NISHIO K, TAKAHASHI M, MIYAHARA Y, TAKEDA Y, KUBOTA N, FUNAYAMA Y, OGASAWARA H, OHIRO T, OHTA S AND SALIO N. (1993). Apoptosis of lung cancer cells caused by some anticancer agents (MMC, CPT-11, ADM) is inhibited by Bcl-2. *Biochem. Biophys. Res. Commun.*, **192**, 30–36.

OLTVAI ZN, MILLIMAN CL AND KORSMEYER SJ. (1993). Bcl-2 heterodimerizes *in vivo* with a conserved homolog, Bax, that accelerates programmed cell death. *Cell*, **74**, 609–619.

PEZZELLA F, TURLEY H, KUZU I, TUNGEKAR MF, DUNNILL MS, PIERCE CB, HARRIS A, GATTER KC AND MASON DY.(1993). Bcl-2 protein in non-small cell lung carcinoma. *N. Engl. J. Med.*, **329**, 690–694.

REED JC. (1994). Bcl-2 and the regulation of programmed cell death. *J. Cell Biol.*, **124**, 1–6.

REED JC, MEISTER L, TANAKA S, CUDDY M, YUM S, GEYER C AND PLEASURE D. (1991). Differential expression of bcl-2 proto-oncogene in neuroblastoma and other tumor cell lines of neural origin. *Cancer Res.*, **51**, 6529–6538.

REEVE JG, MORGAN J, SCHWANDER J AND BLEEHEN NM. (1993). Role for membrane and secreted insulin-like growth factor binding protein-2 in the regulation of insulin-like growth factor action in lung tumors. *Cancer Res.*, **53**, 4680–4685.

TWENTYMAN PR, FOX NE, WRIGHT K AND BLEEHEN NM. (1986). Derivation and preliminary characterisation of adriamycin resistant cell lines of human lung cancer cells. *Br. J. Cancer*, **53**, 529–537.

WALTON MI, WHYSONG D, O'CONNOR PM, HOCKENBERRY D, KORSMEYER SJ AND KOHN KW. (1993). Constitutive expression of human Bcl-2 modulates nitrogen mustard and camptothecin induced apoptosis. *Cancer Res.*, **53**, 1853–1861.

WILLIAMS GT AND SMITH CA. (1993). Molecular regulation of apoptosis: genetic controls on cell death. *Cell*, **74**, 777–779.